# An Unusual Case of Lower Extremity Clear Cell Hidradenocarcinoma

**DOI:** 10.1155/2020/6192109

**Published:** 2020-04-08

**Authors:** Marc Rafols, Odille Mejia, Kei Shing Oh, Blake Bendixen, Irving Jorge, Sumana Narayanan

**Affiliations:** ^1^Department of General Surgery, Mount Sinai Medical Center, Miami Beach, FL 33140, USA; ^2^Department of Pathology, Mount Sinai Medical Center, Miami Beach, FL 33140, USA; ^3^Florida International University Herbert Wertheim College of Medicine, Miami, FL 33199, USA; ^4^Department of Surgical Oncology, Mount Sinai Comprehensive Cancer Center, Miami Beach, FL 33140, USA

## Abstract

Hidradenocarcinoma (HC) is a rare malignant sweat gland tumor with metastatic potential primarily located in the head, neck, and trunk. We present an unusual case of a large lower extremity Clear Cell HC managed with surgical resection and adjuvant locoregional radiation after excluding lymph node involvement.

## 1. Introduction

Clear Cell Hidradenocarcinoma is a very uncommon malignant sweat gland tumor known for high local recurrence rates and lymph node metastasis. Currently, there are no evidence-based guidelines regarding regional lymph node management. The following is a case presentation of Clear Cell HC of the midthigh with inconclusive postoperative PET scan demonstrating mild avidity of an ipsilateral inguinal lymph node that was managed with core needle biopsy, radiation, and close surveillance.

## 2. Case Presentation

**Figure 1:**
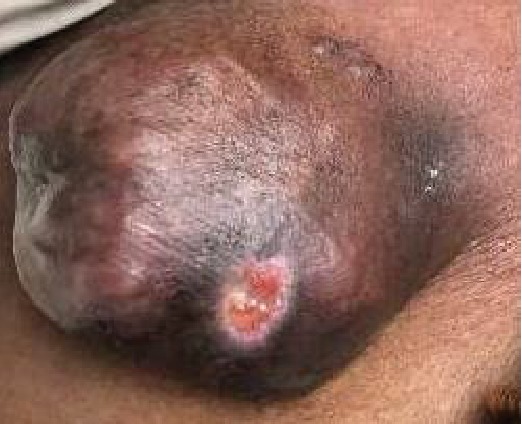
A fungating and draining left anterior upper thigh mass with ulcerations measuring 10 cm in diameter.

**Figure 2:**
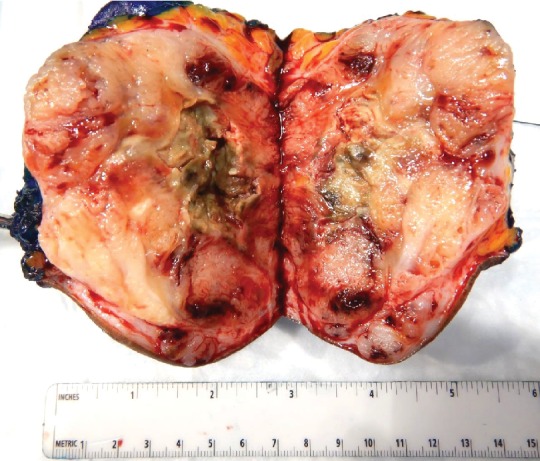
Cut surface shows a well-circumscribed, tan gray, focally necrotic, focally hemorrhagic mass measuring 9 × 9 × 5.5 cm with negative margins, closest margin.

**Figure 3:**
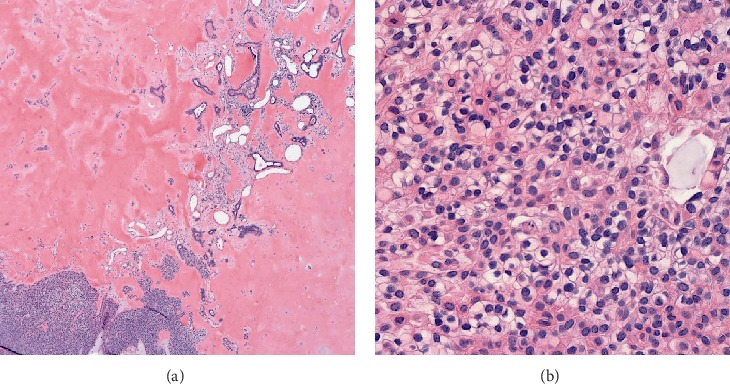
Hidradenocarcinoma arranged in nest and ducts with focal ossification ((a) 2.5 magnification). Mixture of eosinophilic, polygonal, and clear cells with hyperchromatic and enlarged nuclei with prominent nucleoli ((b) 40x magnification).

The patient is a 75-year-old woman with a past medical history of hypertension presented to the office with a 9 × 9 cm fungating left thigh mass that was reported to be slowly growing over the course of two years (Figure 1). She became concerned when it began to drain purulent fluid. She reported a 20 Lb. weight loss over the last few months attributed to decreased appetite. The patient was seen by a general surgeon who performed a wide-margin resection of her tumor. The wound was closed primarily and the patient recovered well from surgery. Pathology returned as Clear Cell Hidradenocarcinoma exhibiting extensive necrosis, hyalinization, focal ossification, and frequent mitoses. All pathologic margins were negative. Immunohistochemical stains were positive for D2-40, p63, CK 5/6, and vimentin. CEA was positive in the ductal component. PAX-8 was negative (Figure 2 and Figure 3).

PET/CT scan was performed after final pathology returned to evaluate for distant metastatic disease. PET/CT demonstrated mildly avid (Max SUV 2.7) 1.1 cm left inguinal and 0.6 external iliac adenopathy. These findings were corroborated with MRI of the pelvis and sonography demonstrating characteristically benign appearing inguinal lymph node. Although this likely represented typical postoperative changes, ultrasound-guided FNA and core needle biopsies of a 2.2 cm inguinal lymph node (without abnormal morphology) were obtained to rule out lymph node metastasis. No other abnormal lymph nodes were identified on ultrasound. Pathology failed to demonstrate any malignant cells on cytology, and immunohistochemistry returned as benign with normal lymph node architecture.

After discussion in multidisciplinary tumor board, the patient was referred to a radiation oncologist and treated utilizing intensity-modulated radiation therapy to a total dose of approximately 5000 cGy in 25 fractions to the lymph nodes and 6400 cGy in 32 fractions to the tumor bed. The patient remains under close surveillance with a follow-up scheduled PET/CT. There is no evidence of recurrence to this date.

## 3. Discussion

Hidradenocarcinoma (HC) is a rare malignant sweat gland tumor with metastatic potential that has the poorest prognosis of all cutaneous carcinomas of eccrine differentiation. Although studies report all-cause 5-year survival of 74% and postsurgical survival of 30%, prognostic studies are limited due to HC's rarity and aggressive nature [1, 2]. Currently, there are no strong consensus guidelines on the management of HC, and most of the information that exists is from limited case series or review articles. HC is incredibly rare, representing approximately 6% of malignant eccrine cancers with an incidence of 0.001% [3].

Eccrine glands are present throughout the entire skin surface with increased concentration in the axilla, hands, and feet [4, 5]. However, the most common area of HC involvement is the scalp (40%) followed by the face (30%) and rarely presents on the extremities [3, 6]. Malignant sweat gland tumors are typically heterogeneous and principally characterized as locally aggressive, demonstrating a high rate of recurrence, up to 10-50% following surgical resection for HC [7]. Despite its aggressive behavior, HC may remain asymptomatic for some time. Often, it presents as either solid or cystic appearing subcutaneous nodules, which may be associated with pruritus or ulceration [1]. HC more frequently arises from de novo mutations as opposed to malignant conversion of benign hidradenomas [8, 9]. Women in the fifth and seventh decade of life are at the greatest risk with a 6 : 4 female : male ratio. HC also has propensity for lymphatic metastasis as well as a propensity towards bone and visceral metastasis [6, 10]. Typical diagnosis is attained via core needle or incisional biopsy with pathological evaluation. Incisional biopsy should be considered over superficial biopsies as the former may potentially only capture the exophytic granulation tissue overlying the tumor and miss the deeper foci of HC [11].

Currently, the recommended approach to management of HC involves wide surgical excision with negative margins as the goal. Mohs micrographic surgery appears to be a possible alternative treatment modality demonstrated with good outcomes and no recurrent disease on some case series [3, 12]. Sentinel lymph node biopsy (SLNB) with preoperative lymphatic mapping may help detect early lymph node metastasis especially if high-risk features are present on clinicopathologic analysis. High-risk features for HC include >2 mm if invasion, perineural invasion, poor differentiation, or location on the ears or lips [10]. Sentinel lymph node biopsy has gained increasing popularity over regional lymph node dissection (LND) in the treatment of cutaneous malignancies as it allows early detection of metastatic disease with less morbidity and may identify patients that would benefit additional systemic therapy. In general, the principles of treatment applied to other cutaneous cancers such as melanoma have been extrapolated to the treatment of eccrine tumors, reserving regional lymph node dissection for patients with clinically positive lymph nodes or those with positive sentinel lymph node biopsy. Limited studies investigating the outcomes of HC cases that use SLNB exist. One study examined five cases of cutaneous eccrine carcinomas that underwent SLNB. Three out of the five patients were found to have metastatic disease in the sentinel node; however, upon completion of lymph node dissection, no additional disease was found. Of the two cases that were HC, one had a positive SLNB with no residual tumor on LND. [13] In our case, a sentinel lymph node biopsy could not be accurately performed given that the tumor had already been completely excised at the time of clinicopathologic diagnosis. Previous studies have also noted that postsurgical changes may mimic suspicious lymphadenopathy on PET/CT by demonstrating reactive avidity [14]. This is likely the case with our patient given lack of suspicious adenopathy on postoperative ultrasound and negative core needle biopsy.

Currently, no clear recommendations exist regarding the best postsurgical management of HC. Chemotherapy and radiation therapy have been reported for cases of metastatic HC [3, 15]. Although radiotherapy has been frequently applied to the treatment of HC with some conflicting results, some cases have shown a good response while others have demonstrated radio resistance [12, 16–19]. Adjuvant chemotherapy has primarily consisted of 5 FU/capecitabine as first line treatment, doxorubicin, platins or cyclophosphamide, vincristine, and bleomycin as second line; however, studies have failed to demonstrate a clear survival benefit [20]. Combination chemotherapy and radiation has also not shown a clear survival advantage or effect on local control [16]. Targeted therapy such as trastuzumab has shown stabilized disease progression in tumors with HER2/neu expression [20]. In a few cases of metastatic HC with estrogen receptor expression, tamoxifen has also demonstrated lymph node regression and palliation of bone metastasis [21]. Considering that HC has a high rate of locoregional recurrence even with negative margin resection, there is no clear data evaluating which patients would benefit from radiation to the surgical bed or the draining lymph node basin. Given our patient preference and multidisciplinary tumor board consensus, we proceeded with adjuvant radiation of the surgical bed and inguinal lymph node basin given the large tumor size and the proximity of the deep margin.

## 4. Conclusion

HC is a rare but aggressive eccrine gland tumor that rarely presents in the extremities. Currently, no clear evidence-based guidelines exist especially for patients with questionable lymph node involvement. Our case of a large thigh HC presents the clinical dilemma of interpreting postexcision PET scan with mild avidity in the regional lymph node basin. In our case, the suspicious lymph node was managed by core needle biopsy to assess for lymph node involvement followed by adjuvant radiotherapy to the primary tumor site and inguinal lymph node basin. Close clinical follow-up studies show no evidence of locoregional recurrence or distant disease. The use of sentinel lymph node biopsy and radiotherapy in cases of HC remains controversial.
